# A Novel Thin-Layer Flow Cell Sensor System Based on BDD Electrode for Heavy Metal Ion Detection

**DOI:** 10.3390/mi15030363

**Published:** 2024-03-04

**Authors:** Danlin Xiao, Junfeng Zhai, Zhongkai Shen, Qiang Wang, Shengnan Wei, Yang Li, Chao Bian

**Affiliations:** 1 State Key Laboratory of Transducer Technology, Aerospace Information Research Institute, Chinese Academy of Sciences, Beijing 100190, China; xiaodanlin1998@163.com (D.X.); weishengnan11@163.com (S.W.); 2 School of Electronic, Electrical and Communication Engineering, University of Chinese Academy of Sciences, Beijing 100049, China; 3 State Key Laboratory of Electroanalytical Chemistry, Changchun Institute of Applied Chemistry, Chinese Academy of Sciences, Changchun 130022, China; jfzhai@ciac.ac.cn; 4 Hebei Sailhero Environmental Protection High-Tech Co., Ltd., Shijiazhuang 050036, China; shenzhongkai1987920@126.com (Z.S.); xhwq7809@126.com (Q.W.)

**Keywords:** thin-layer flow cell, heavy metal ion, boron-doped diamond electrode, automatic detection system, electrochemical sensor

## Abstract

An electrochemical sensor based on a thin-layer flow cell and a boron-doped diamond (BDD) working electrode was fabricated for heavy metal ions determination using anodic stripping voltammetry. Furthermore, a fluidic automatic detection system was developed. With the wide potential window of the BDD electrode, Zn^2+^ with high negative stripping potential was detected by this system. Due to the thin-layer and fluidic structure of the sensor system, the electrodepositon efficiency for heavy metal ions were improved without using conventional stirring devices. With a short deposition time of 60 s, the system consumed only 0.75 mL reagent per test. A linear relationship for Zn^2+^ determination was displayed ranging from 10 μg/L to 150 μg/L with a sensitivity of 0.1218 μA·L·μg^−1^ and a detection limit of 2.1 μg/L. A high repeatability was indicated from the relative standard deviation of 1.60% for 30 repeated current responses of zinc solution. The system was applied to determine Zn^2+^ in real water samples by using the standard addition method with the recoveries ranging from 92% to 118%. The system was also used for the simultaneous detection of Zn^2+^, Cd^2+^, and Pb^2+^. The detection results indicate its potential application in on-site monitoring for mutiple heavy metal ions.

## 1. Introduction

With the advancement in industrialization and technology, a large amount of industrial wastewater containing heavy metal ions is generated and discharged into the environment [1]. Heavy metal ions discharged into the environment threaten ecosystem stability and human health with the characteristics of high toxicity, biological enrichment, and non-biodegradability [2]. Lead ions can cause damage to the human nervous, immune, hematopoietic, reproductive, and digestive systems, while excess cadmium ions can lead to severe bone damage [3]. Although zinc ions are one of the most important trace elements in the body, excess intake may cause a series of diseases such as anemia, epigastric pain, Alzheimer’s disease, Parkinson’s disease, diabetes, and cancer [4,5].

At present, analytical methods for detecting heavy metal ions mainly include atomic absorption spectrometry (AAS) [6,7], inductively coupled plasma mass spectrometry (ICP-MS) [8,9,10], and fluorescent probes [11]. These methods are usually carried out in laboratory environments and are difficult to be implemented to the portable rapid detection due to requiring bulky expensive instruments, sophisticated operations, and long analysis time. Electrochemical methods have gained attention due to their low cost, rapid analysis, and high sensitivity [12]. Anodic stripping voltammetry (ASV) is a commonly used electrochemical method for trace detection of heavy metal ions [13,14,15,16,17].

ASV involves two steps. Firstly, a certain voltage is applied to the working electrode to deposit the target ions on its surface by reduction. Then, a reverse scan voltage is applied to dissolve the deposited targets, obtaining a stripping current related to the concentration of heavy metal ions and achieving precise detection [18,19,20]. As the main area for electrochemical reactions, the working electrode plays a crucial role in influencing the detection performance of electrochemical sensors. The heavy metal ions that can be detected are influenced by the potential window of the working electrode. Conventional electrodes such as the gold electrode and platinum electrode have a narrow potential window, which makes it difficult to detect strongly electronegative heavy metal ions, such as zinc ions. During the detection of zinc ions, the electrodes may generate hydrogen evolution bubbles, which prevent the accumulation of zinc ions on the surface of the electrode and affect the detection results. Boron-doped diamond (BDD) electrodes have a wide potential window, ranging from approximately −2 V to +1 V [21,22]. This characteristic provides the possibility for the detection of Zn^2+^ with high negative stripping potential, as well as the simultaneous detection of multiple ions. Furthermore, BDD electrodes exhibit excellent characteristics, including low and stable background currents and resistance to electrode contamination for the detection of heavy metal ions, making them ideal electrochemical electrodes for heavy metal ions [23,24,25].

In traditional electrochemical analysis for heavy metal ions, stirring is often employed during the deposition step to enhance the enrichment efficiency and achieve trace detection [26]. The addition of a stirring device increases the complexity of the equipment. In addition, variations in parameters like stirrer intensity, speed, and time may affect the rate and uniformity of ion deposition on the electrode surface. To improve the detection limit and enhance the analytical performance of the sensor, most of the studies focused on the sensitive materials such as introducing nanomaterials on the electrode surface [17,27,28,29]. Extending the deposition time is also an approach, but it increases the detection time.

This work presents the development of an electrochemical sensor system for heavy metal ions determination. The developed system utilizes a thin-layer electrochemical flow cell and an automatic flow system, which allows for a flow-based process instead of stirring. During the deposition process, the fresh sample continuously flows over the electrode surface, which improves the efficiency of the electrodeposition and reduces the detection time. In addition, the stable flow rates can minimize the impact of external factors on measurement results, enhancing the stability of the system. The working electrode used is BDD, which enables the detection of multiple ions. The thin-layer flow cell, with a height of 300 μm and a volume of 20 μL, is used as an alternative to a traditional beaker for the electrolytic cell. Only 0.75 mL of sample is required per test, which reduces sample consumption and minimizes secondary pollution. This design provides convenience for conducting on-site portable detection of heavy metal ions.

## 2. Materials and Methods

### 2.1. Material and Reagents

Potassium chloride (KCl, AR, 99.5%), sodium acetate anhydrous (CH_3_COONa, AR), and glacial acetic acid (CH_3_COOH, AR) were purchased from Sinopharm Chemical Reagent Co. (Shanghai, China). Standard solutions (1000 μg/mL) of Zn, Pb, Cd, and Bi were purchased from the Institute for Environment Reference Materials Ministry of Environmental Protection (Beijing, China). All aqueous solutions used in this work were prepared with highly pure deionized water (Milli-Q system Millipore Company, Darmstadt, Germany) with a resistivity of 18 MΩ·cm.

The reserve solutions of zinc (Zn), lead (Pb), cadmium (Cd), and bismuth (Bi) at a concentration of 10 µg/mL were prepared by diluting their standard solutions of 1000 µg/mL with deionized water. Test samples, containing varying concentrations of target ions, were prepared using the reserve solutions and a supporting electrolyte solution. The supporting electrolyte solution consisted of 0.3 mol/L KCl and 0.1 mol/L acetic acid buffer. The pH of the buffer solution was adjusted to 4.5 with glacial acetic acid.

Real water samples for analysis were collected from several ponds in Yuanmingyuan and prepared with the same concentrations of supporting electrolytes.

### 2.2. Apparatus

A Gamry Reference 600 electrochemical workstation (Gamry Instruments, Warminster, PA, USA) was used to optimize detection parameters. A Runze SY03B-M07 multi-channel syringe pump (Runze Fluid, Nanjing, China), HY-E100X portable electrochemical workstation (Haoyang Technology, Shenzhen, China) and XHBX9100 portable water quality multi-parameter analyzer software V1.0.0 (Hebei Sailhero Environmental Protection High-Tech Co., Ltd., Shijiazhuang, China) were used for the construction of the fluidic automatic detection system. The three-electrode system of the developed electrochemical sensor included a planar BDD electrode as working electrode, a platinum wire as counter electrode, and an Ag/AgCl as reference electrode (CHI 111).

### 2.3. Design of the Thin-Layer Flow Cell

**Figure 1:**
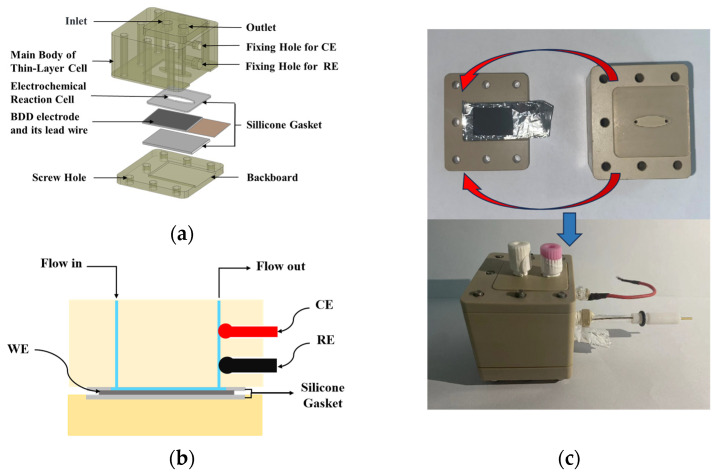
(**a**) The three-dimensional structure diagram; (**b**) the cross-sectional diagram; (**c**) photograph of the thin-layer flow cell.

The thin-layer flow cell integrates the three-electrode system, fluid channel, and electrochemical reaction cell. The diagram and the photograph of the thin-layer flow cell are shown in Figure 1. As shown in Figure 1a, the cell mainly consists of the main body and the back plate. The main body of the thin-layer cell is designed with an inlet channel; an outlet channel; an electrochemical reaction cell; and two fixing holes for reference electrode and counter electrode, respectively. As shown in the cross-sectional drawing of the thin-layer flow cell in Figure 1b, the working electrode is secured between the main body of the thin-layer cell and the back plate with two silicone gaskets. The back plate provides the mechanical strength and stability to support and protect the working electrode. The water sample enters the thin-layer cell through the inlet, flows through the fluid channel, passes the surface of the working electrode, and then exits through the outlet. The electrochemical reaction cell has a height of 300 µm and a volume of 20 µL, which reduces the sample consumption compared with the traditional electrochemical methods.

These components are connected and secured in place using screws, ensuring a robust connection while also allowing for easy disassembly for maintenance and replacement of electrodes and other equipment.

### 2.4. Design of the Fluidic Automatic Detection System

**Figure 2:**
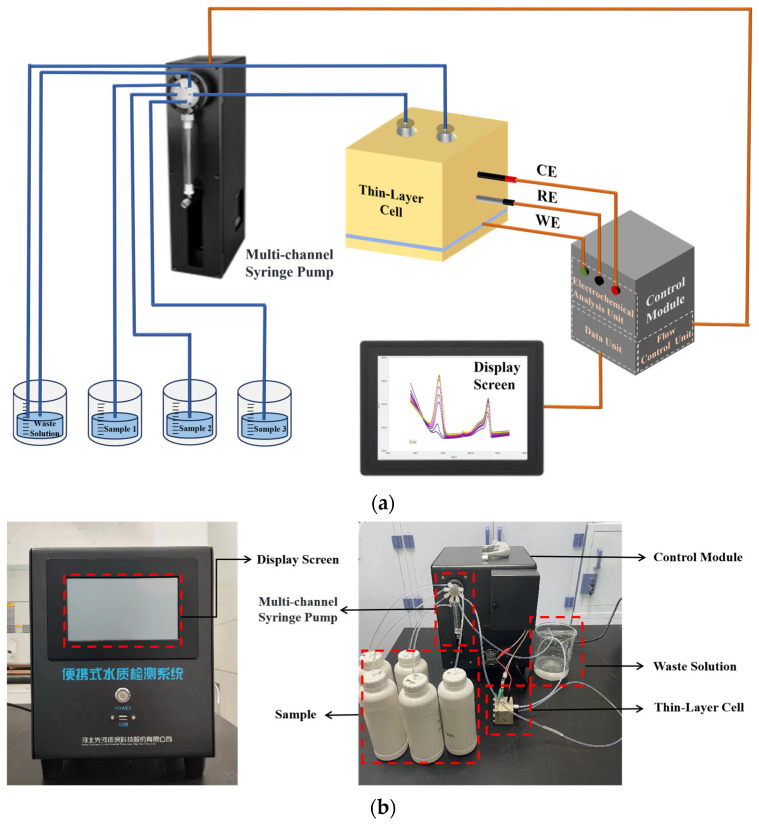
(**a**) Schematic diagram and (**b**) the photograph of the developed automated detection system.

The detection system was produced based on the thin-layer flow cell and the automatic flow system. As shown in Figure 2a, the fluidic automatic detection system consists of four main components: the thin-layer flow cell, a multi-channel syringe pump, a control module, and a display screen. The thin-layer flow cell is the primary component. The multi-channel syringe pump is intergrated with a multiport selector valve for controlling the switching of multiple channels and the flow rate. The control module consists of the flow control unit, electrochemical analysis unit, and data unit. The flow control unit manages the multiport selector valve, allowing the syringe pump to gradually introduce the required samples into the electrochemical reaction cell. By controlling the number of steps the syringe pump makes per unit time, the flow rate of the sample can be adjusted. The electrochemical analysis unit applies potential to the three-electrode system, controls the electrochemical detection method, and detects the current response of the electrochemical sensor. The data unit is used for data collection, storage, and processing. The display screen is used to show the tested curves and detection results in real time. The photograph of the developed automated detection system is shown in Figure 2b. The detection system, with a size of 25.5 cm × 27.5 cm × 29 cm, can be used for on-site determination.

### 2.5. Pretreatment for the BDD Electrode

The BDD electrode was cleaned prior to its first use to remove surface impurities. The cleaning process involves soaking the electrode in acetone for 10 min, followed by sonication in ethanol for 5 min, and finally sonication in deionized water for 5 min. In addition, before the first use, the surface of the BDD electrode was subjected to anode pretreatment and cathode pretreatment in turn to clean and activate the electrode surface. During anode pretreatment, a 3 V constant potential is applied for 360 s in 0.5 mol/L H_2_SO_4_. For cathode pretreatment, a −3 V constant potential is applied for 360 s in 0.5 mol/L H_2_SO_4_. After pretreatment, cyclic voltammetry (CV) scans should be performed daily before use, with a range from −0.8 V to 0.2 V, to ensure proper functioning of the BDD electrode.

### 2.6. Experimental Procedures

**Figure 3:**
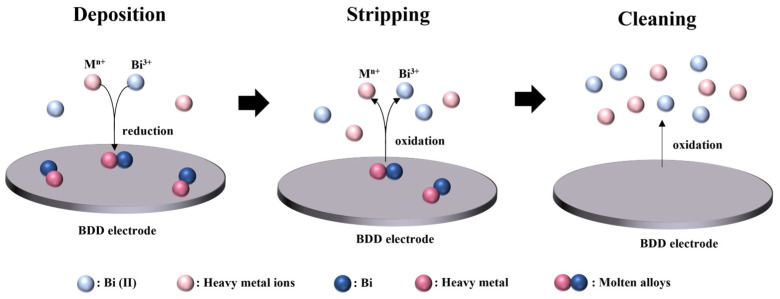
The electrochemical process for heavy metal ions detection.

The electrolyte containing Bi (II) and target heavy metal ions were injected into the thin-layer cell through the multi-channel syringe pump. The ASV test was conducted, and the electrochemical process is shown in Figure 3. The heavy metal ions were first electrodeposited on the surface of the BDD electrode under a certain voltage by reduction. Bi ions can form molten alloys with target heavy metal ions, enhancing the current response [30]. During the deposition process, the sample flowed through the thin-layer cell and the electrode surface at a constant flow rate. Then, the heavy metal ions were dissolved within the proper potential window. During the stripping process, the sample remained stationary. Prior to the next test, the cleaning step was carried out to eliminate residual heavy metal ions from the working electrode by applying a high voltage.

## 3. Results and Discussion

### 3.1. Effect of KCl Concentration

**Figure 4:**
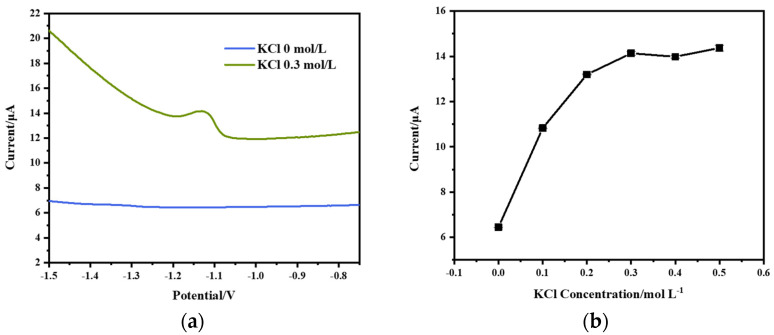
Effect of KCl concentration on the detection of Zn^2+^: (**a**) square wave voltammogram curves of Zn^2+^ (200 μg/L) in acetate buffer with KCl and without KCl; (**b**) effect of KCl concentration on stripping peak current. Reaction conditions: deposition potential: −1.6 V, deposition time: 90 s, frequency: 25 Hz, amplitude: 50 mV, step potential: 5 mV, flow rate: 0.3 mL/min.

In this study, acetic acid–sodium acetate buffer (0.1 mol/L, pH = 4.5) was used as the supporting electrolyte solution. The effect of the addition of KCl in the buffer on the stripping peak current of Zn^2+^ (200 µg/L) was investigated. As shown in Figure 4a, the addition of KCl to the buffer solution resulted in a significant enhancement of the stripping peak signal compared to the KCl-free buffer solution. In the absence of KCl, the diffusion resistance of the thin-layer cell is large, resulting in a great ohmic drop. This reduces the driving force for electron transfer at the electrode surface [31], making it difficult to achieve the desired current response. The addition of KCl to the buffer solution increases the conductivity of the electrolyte solution, reducing the ohmic drop and improving detection performance. It has been reported [32] that Zn^2+^ forms weak complexes with acetate and chloride ions, which facilitates the reduction and increases the stripping peak signal. Consequently, KCl was incorporated into the acetic acid buffer solution as the supporting electrolyte solution in subsequent experiments.

In the range of 0–0.5 mol/L, the effect of the concentration of KCl in the supporting electrolyte solution on the stripping peak current of Zn^2+^ (200 µg/L) was investigated. As shown in Figure 4b, the stripping peak current increased gradually with the concentration of KCl. However, the growth rate of the stripping peak current was slowed down when the concentration of KCl was higher than 0.3 mol/L. The reason for this is that as the concentration of KCl is excessively high, the adsorption of ions on the surface of the electrode can lead to a capacitance effect that generates the non-Faradaic current [33].

### 3.2. Effect of the Concentration of Bismuth Ion

Bismuth and mercury can form molten alloys with zinc ions, which facilitate the stripping processes of Zn^2+^ on the electrode surface [30]. This molten alloy enhances the current response, resulting in more sensitive and accurate measurement. Bi is known for its environmentally friendly nature and low toxicity, making it a subject of widespread attention in practical applications. The use of Bi for heavy metal detection results in highly reproducible stripping responses and clear peak shapes. This excellent peak separation performance enables precise and reliable analysis of adjacent peaks. In addition, the bismuth film is insensitive to dissolved oxygen. Therefore, it is unaffected by oxygen during the reaction process, resulting in a high sensitivity and a wide linear detection range.

Two methods are commonly used for Bi deposition: in situ deposition and ex situ deposition. In situ deposition has several advantages over ex situ deposition. These include no additional treatment, ease of operation, cost savings, and streamlined experimental procedures [34]. Therefore, the convenient and efficient in situ deposition method was chosen in this study.

**Figure 5:**
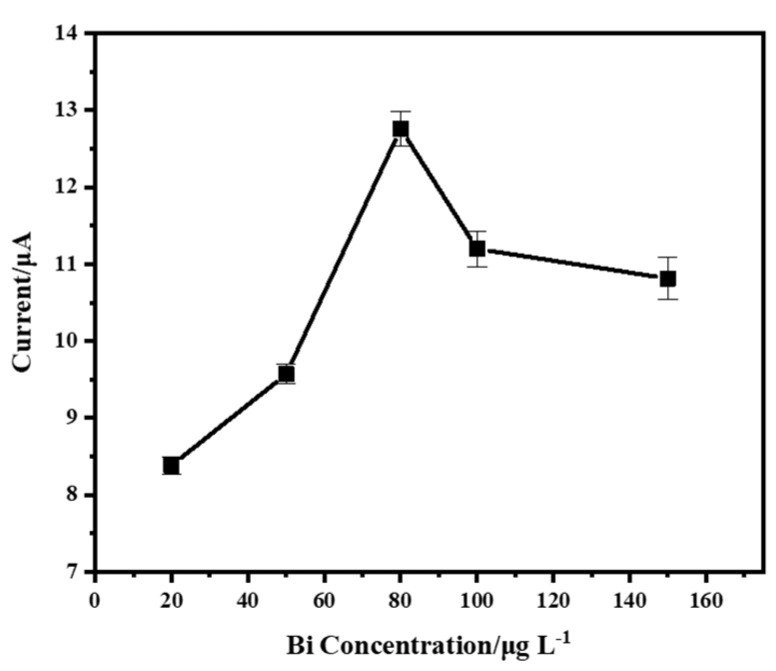
Effect of Bi concentration on stripping peak current. Reaction conditions: deposition potential: −1.6 V, deposition time: 90 s, frequency: 25 Hz; amplitude: 50 mV, step potential: 5 mV, flow rate: 0.3 mL/min.

The effect of Bi concentration on the stripping peak current of Zn^2+^ (200 µg/L) was investigated. The results shown in Figure 5 indicate that the stripping peak current of Zn^2+^ increased with the increase in Bi concentration in the range of 20 µg/L to 80 µg/L. Bi facilitates the deposition and stripping processes of Zn^2+^ on the electrode surface, increasing the stripping peak signal. However, a gradual decrease in the peak current was subsequently observed, when the concentration of Bi exceeded 80 µg/L. The reason for this is that the high concentration of Bi ions competes with Zn ions, occupying the active sites and preventing the accumulation and stripping of Zn ions. A concentration of 80 µg/L was chosen for the in situ deposition solution of Bi for the following experiments.

### 3.3. Effect of Flow Rate

**Figure 6:**
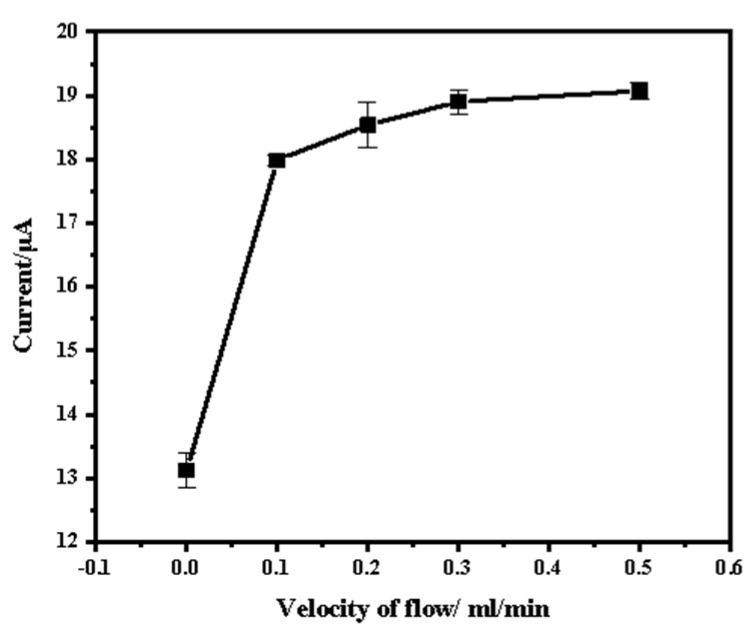
Effect of flow rate on stripping peak current. Reaction conditions: deposition potential: −1.8 V; deposition time: 60 s; frequency: 25 Hz; amplitude: 100 mV; step potential: 5 mV.

In this detection system, the sample flows during the deposition and cleaning process to achieve high deposition efficiency and remove bubbles from the surface of the working electrode. This process is similar to the conventional stirring process using a magnetic stirrer. Therefore, the flow rate is a crucial parameter that significantly affects the current response and the sensitivity of the detection system. During the stripping process, the sample remains stationary to produce stable current responses. The effect of flow rate on the stripping peak current of Zn^2+^ (200 µg/L) was investigated in the range of 0 mL/min to 0.5 mL/min. As shown in Figure 6, as the flow rate was increased, the stripping peak current also increased gradually. This could be attributed to the acceleration of the ion transfer rate and the increase in the deposition of the Zn ion. Once the flow rate exceeded 0.3 mL/min, the increasing trend became less pronounced. This may be due to the electrode surface gradually becoming saturated. It is important to note that higher flow rates require more sample consumption, which may cause secondary contamination to the environment. A flow rate of 0.3 mL/min was selected for subsequent experiments.

### 3.4. Effect of SWV Pulse Amplitude

**Figure 7:**
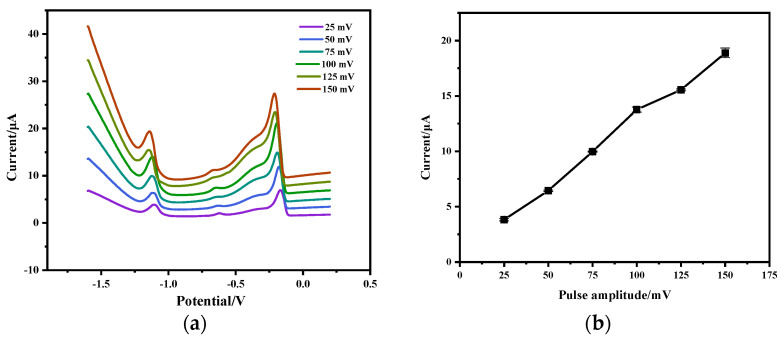
Effect of SWV pulse amplitude on the detection of Zn^2+^: (**a**) square wave voltammogram curves of Zn^2+^ (200 μg/L) with different pulse size; (**b**) effect of pulse amplitude on stripping peak current. Reaction conditions: deposition potential: −1.6 V, deposition time: 90 s, frequency: 25 Hz, step potential: 5 mV, flow rate: 0.3 mL/min.

Square Wave Voltammetry (SWV) pulse size on the stripping peak current of Zn^2+^ (200 µg/L) was investigated in the range of 25 mV–150 mV. As shown in Figure 7b, the stripping peak current consistently increases with pulse amplitude. However, as shown in Figure 7a, the baseline of the stripping voltammogram also increases accordingly. A pulse amplitude of 100 mV was chosen for subsequent experiments.

### 3.5. Effect of Deposition Potential and Deposition Time

**Figure 8:**
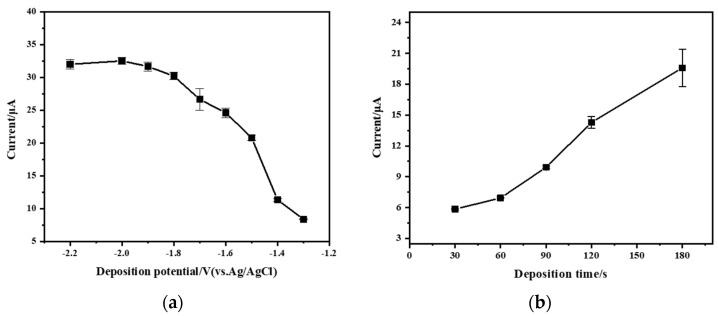
(**a**) Effect of deposition potential on stripping peak current. Reaction conditions: deposition time: 90 s; frequency: 25 Hz; amplitude: 50 mV; step potential: 5 mV; flow rate: 0.3 mL/min. (**b**) Effect of deposition time on stripping peak current. Reaction conditions: deposition potential: −1.8 V, frequency: 25 Hz, amplitude: 100 mV, step potential: 5 mV, flow rate: 0.3 mL/min.

The deposition potential is a crucial factor that affects the stripping peak current. In this study, the effect of the deposition potential on the stripping peak current of Zn^2+^ (200 µg/L) was investigated in the range of −2.2 V to −1.3 V. As shown in Figure 8a, when the deposition potential shifted negatively from −1.3 V to −1.8 V, the stripping peak current increased significantly. This phenomenon may be attributed to the fact that, within this potential range, the electron driving force on the electrode surface increases as the accumulation potential gradually becomes negative. Consequently, the zinc ions reduced to the working electrode surface also increase. The stripping peak current exhibited a slow increase, or even a slight decrease, as the deposition potential shifted negatively from −1.8 V to −2.2 V. The reason may be that the excessively negative deposition potential causes the generation of hydrogen gas, affecting the deposition of Zn^2+^. Additionally, the zinc ions deposition on the surface of the working electrode may have reached a saturated state [12]. Therefore, a deposition potential of −1.8 V was used for subsequent experiments.

Figure 8b shows the effect of deposition time (30 s to 120 s) on the stripping peak current for Zn^2+^ (200 µg/L). The results show that peak current gradually increased with deposition time. However, extending the deposition time excessively would result in longer detection time and lower detection efficiency. In addition, the linear range may be shortened and the accuracy of the detection may be compromised if the deposition time is too long. Therefore, a deposition time of 60 s was chosen for subsequent experiments.

### 3.6. Effect of Cleaning Potential and Cleaning Time

**Figure 9:**
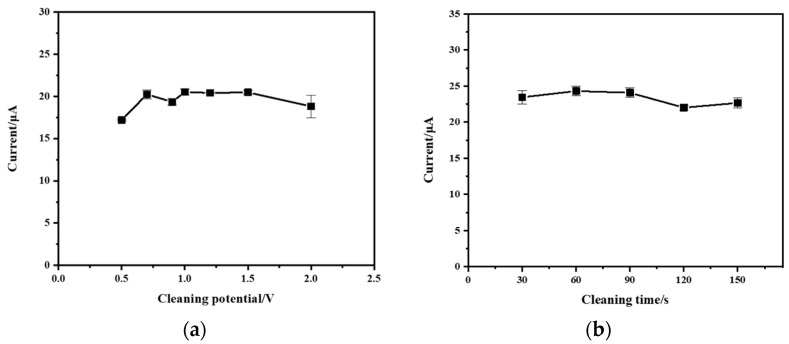
(**a**) Effect of cleaning potential on stripping peak current. Reaction conditions: deposition potential: −1.8 V; deposition time: 60 s; frequency: 25 Hz; amplitude: 100 mV; step potential: 5 mV; flow rate: 0.3 mL/min. (**b**) Effect of cleaning time on stripping peak current. Reaction conditions: deposition potential: −1.8 V; deposition time: 60 s; frequency: 25 Hz; amplitude: 100 mV; step potential: 5 mV; flow rate: 0.3 mL/min.

After the stripping process, the cleaning potential is applied to eliminate residual heavy metal ions from the working electrode. The effect of the cleaning potential on the stripping peak current of Zn^2+^ (200 µg/L) was investigated in the range of 0.5 V to 2 V. As shown in Figure 9a, there was no significant change in the magnitude of the stripping peak current under different cleaning potentials. This suggests that within the range of 0.5 V to 2 V, different cleaning potentials did not significantly affect the stripping process of zinc ions. However, lower potentials may not completely remove the residual zinc ions on the working electrode surface, causing interference in subsequent experiments. On the other hand, higher potentials may lead to oxygen evolution, affecting the cathodic protection of the BDD electrode. This may be the reason for the large error when the cleaning potential of 2 V is applied. Taking all of these factors into consideration, a cleaning potential of 1 V was selected for the following experiments.

The effect of cleaning time on the stripping peak current of Zn^2+^ (200 µg/L) was investigated between 25 s and 150 s. Figure 9b shows that the stripping peak current was not significantly affected by cleaning time between 25 s and 150 s. Despite this, a longer cleaning time can effectively remove residual heavy metals from the surface of the working electrode and enhance the accuracy of the experiment. Considering both the cleaning effectiveness and experimental efficiency, a cleaning time of 90 s was selected.

### 3.7. Analysis of Sensing Performance

**Figure 10:**
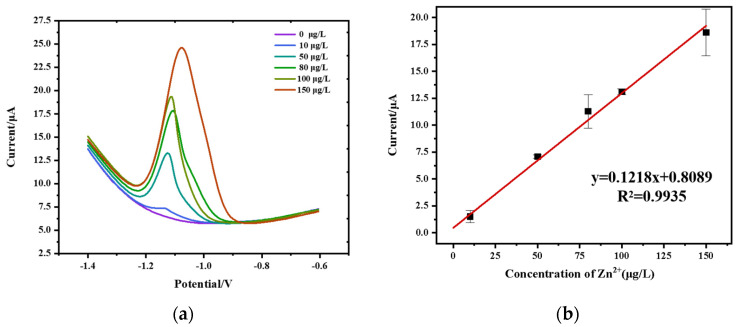
(**a**) SWV curves; (**b**) calibration curve for Zn^2+^ determination.

The performance of the sensor system for Zn^2+^ determination was tested under the optimized conditions, and 0.75 mL of reagent is consumed for each test. Stripping voltammetry curves for the detection of Zn^2+^ are displayed in Figure 10a. The relationship between the concentration of Zn^2+^ and the corresponding peak current is shown in Figure 10b. The system showed a good linear relationship for Zn^2+^ determination in the range from 10 μg/L to 150 μg/L (R^2^ = 0.9935) with a sensitivity of 0.1218 μA·L·μg^−1^. The limit of detection (LOD) was estimated according to the linear equation by the following equation:

$$LOD=\frac{3\sigma }{S}$$

Here, *σ* represents the standard deviation of the response current obtained from the blank buffer solution, while *S* denotes the sensitivity, which corresponds to the slope of the linear equation. The calculated LOD value was determined to be 2.1 μg/L.

**Figure 11:**
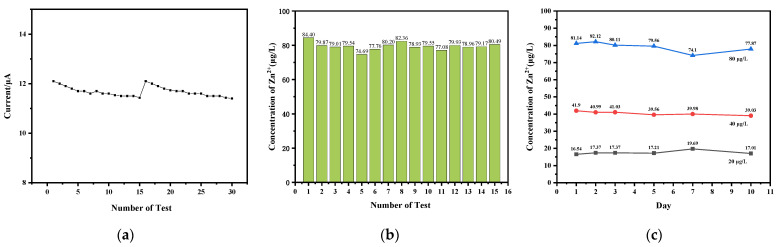
(**a**) The response current in 80 μg/L Zn(II) for 30 consecutive tests; (**b**) the detection results in 80 μg/L Zn(II) for 15 consecutive tests; (**c**) the detection results in 20 μg/L, 40 μg/L, and 80 μg/L Zn(II) for the long-term (10 d) stability tests.

The repeatability and long-term stability of the sensor system were evaluated and demonstrated. The repeatability was evaluated using an 80 μg/L Zn^2+^ solution. The results of 30 consecutive tests are shown in Figure 11a. The average response current was determined to be 11.67 μA, with a relative standard deviation (RSD) of 1.6%. Multiple calibration tests were conducted using an 80 μg/L Zn^2+^ solution. A total of 15 tests were performed. Figure 11b presents the results of the Zn^2+^ concentration tests. The concentrations of Zn^2+^ measured ranged from 74.69 μg/L to 84.40 μg/L, with an RSD of 2.75%. The response current and measured concentration showed minimal variation and insignificant deviations during repeated tests at the same concentration. Furthermore, the sensor system demonstrates excellent long-term stability for ten days. The detection results in 20 μg/L, 40 μg/L, and 80 μg/L Zn(II) are shown in Figure 11c with RSDs of 6.2%, 2.6%, and 3.6%, respectively, for long-term (10 d) stability tests. These results indicate that the automated detection system has good repeatability and long-term stability.

Analytical performance comparisons between the developed sensor system and other reported electrochemical sensors for the detection of Zn^2+^ ions are shown in Table 1. The sensor system developed was found to have sufficient sensitivity, short deposition time, and excellent repeatability. These good performance characteristics of the developed sensor system can be attributed to the exceptional properties of the BDD electrode material, as well as the use of the thin-layer cell and the automated flow system. The fluidic thin-layer cell improved the contact and reaction efficiency between the working electrode and the sample. This is because the fresh sample, with the original concentration of target substances, flows continuously through the electrode surface during the deposition process. Therefore, it is possible to enhance the deposition of target substances, improve the response current, and shorten the detection period. In addition, the stable flow rates and conditions of the electrochemical system can reduce the influence of external factors on measurement results, which could enhance the stability and reliability of the sensor system.

### 3.8. Real Sample Detection

The standard addition method was conducted to evaluate the performance of the developed sensor system for Zn^2+^ detection in practical applications. Real water samples were taken randomly from three ponds in Yuanmingyuan. As shown in Table 2, the recovery values from the detection of Zn^2+^ were in the range from 92% to 118%.

### 3.9. Simultaneous Detection of Pb^2+^, Cd^2+^ and Zn^2+^

**Figure 12:**
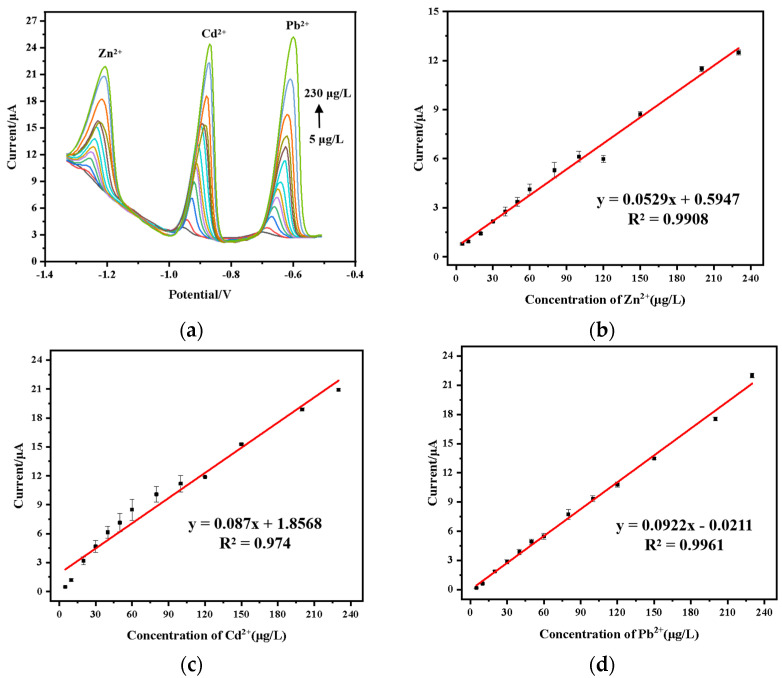
Simultaneous detection of Zn^2+^, Cd^2+^, and Pb^2+^: (**a**) stripping voltammetry curves; (**b**) calibration curve for Zn^2+^; (**c**) calibration curve for Cd^2+^; (**d**) calibration curve for Pb^2+^.

The wide potential window of the BDD electrode enables simultaneous detection of multiple heavy metal ions. In this experiment, three common heavy metal ions—Pb^2+^, Cd^2+^, and Zn^2+^—were detected with the sensor system developed. As shown in Figure 12, the linear relationships between the stripping peak current and the concentration of Zn^2+^, Cd^2+^, and Pb^2+^ are displayed within the range of 5 μg/L to 230 μg/L. The sensitivities for the detection of Zn^2+^, Cd^2+^, and Pb^2+^ were 0.0529 μA·L·μg^−1^, 0.0870 μA·L·μg^−1^, and 0.0922 μA·L·μg^−1^, respectively. The LODs were determined to be 0.80 μg/L, 0.53 μg/L, and 0.17 μg/L. Compared with the individual detection of Zn^2+^, the sensitivity for simultaneous detection of multiple ions was lower. This may be due to the interaction and competition between the heavy metal ions. In addition, the peak potential shifted in a positive direction, possibly due to the interaction forces between deposited metal particles [42].

## 4. Conclusions

An electrochemical sensor based on a thin-layer flow cell and a boron-doped diamond (BDD) working electrode was fabricated for heavy metal ions determination. Further, a fluidic automatic detection system was developed. The constructed sensor system was employed to detect Zn^2+^, demonstrating good linearity within the range of 10–150 μg/L, with a sensitivity of 0.1218 μA·L·μg^−1^ and a detection limit of 2.1 μg/L. The deposition time was 60 s, which reduced the test time sharply. A 0.75 mL sample was consumed by this sensor system during each test, which greatly reduced the reagent consumption and the risk of secondary pollution to the environment. The RSD of the current responses obtained from 30 repeated tests was 1.6%. The recoveries for real water sample analysis by using standard addition method were in the range from 92% to 118%. Furthermore, the system was used to achieve the simultaneous detection of multiple heavy metal ions. The linear range for simultaneous detection of Zn^2+^, Cd^2+^, and Pb^2+^ was from 5 μg/L to 230 μg/L, with LODs of 0.80 μg/L, 0.53 μg/L, and 0.17 μg/L, respectively. These experimental results indicate that the developed automated detection system enabled sensitive and stable testing of heavy metal ions, showing potential for on-site monitoring applications. In order to improve the analytical performance of the sensor system for the simultaneous detection of multiple heavy metal ions, the parameters (e.g., Bi concentration, deposition potential, and deposition time) should be further studied and optimized in future work.

## Figures and Tables

**Table 1:** Comparison of analytical performances of the developed electrochemical sensor system with other reported sensors for the detection of Zn ions.

Electrode Type	LOD (μg/L)	Sensitivity (μA·L·μg^−1^)	Linear Range (μg/L)	Deposition Time (s)	RSD (%)	Reference
PEDOT/PVA/AgNPs/SPCE	6.000	0.041	10–80	240	-	[27]
Ti_3_C_2_T_x_/MWNTs/Au	1.500	0.040	200–600	120	-	[28]
AuNPs/PANI-MWCNTs/SPCE	0.039	0.619	1–180	400	3.48	[29]
Bi/screen-printed gold electrode	0.050	-	1–120	180	2.05	[35]
BDD	13.00	0.060	33–1300	10	5.60	[36]
Bi film electrode/glassy carbon electrode	1.070	0.091	5–110	120	4.74	[37]
Hg-Bi/PDAAQ/GC	0.169	7.342	0.1–100	300	4.74	[38]
Bi/graphene oxide/glassy carbon electrode	6.000	0.402	20–8000	480	8.70	[39]
Bi/bismuth and graphdiyne/GCE	0.010	-	0.065–65	150	2.03	[40]
Ex-situ Bi/Nafion/glassy carbon electrode	2.300	0.045	2.5–500	360	11.9	[41]
BDD	2.100	0.122	10–150	60	1.60	This work

PEDOT/PVA/AgNPs/SPCE: poly (3,4-ethylenedioxythiophene)/poly (vinyl alcohol)/silver nanoparticles/screen-printed carbon electrode; Ti_3_C_2_T_x_/MWNTs/Au: titanium carbide/multiwalled carbon nanotubes/gold electrode; AuNPs/PANI-MWCNTs/SPCE: gold nanoparticles/polyaniline-multi-walled carbon nanotubes composite/screen-printed carbon electrode; Hg-Bi/PDAAQ/GC: bimetallic film of mercury and bismuth/poly(1,2-diaminoan-thraquinone)/glassy carbon electrode.

**Table 2:** Results of the standard addition experiment for Zn^2+^ determination in real water samples.

Sample	Added (μg/L)	Detected (μg/L)	Recovery (%)
Water 1	0	-	-
	80	85.14	106
	120	141.39	117
	200	214.86	107
Water 2	0	-	-
	80	87.15	108
	100	91.34	92
	120	125.40	104
Water 3	0	-	-
	50	53.38	106
	80	90.64	113
	120	141.88	118

## Data Availability

The data that support the findings of this study are available from the corresponding authors upon reasonable request.
